# Investigation and Application of Key Alignment Parameters for Overlay Accuracy in 3D Structures

**DOI:** 10.3390/mi16080876

**Published:** 2025-07-29

**Authors:** Miao Jiang, Mingyi Yao, Ganlin Song, Yuxing Zhou, Jiani Su, Yuejing Qi, Jiangliu Shi

**Affiliations:** 1 Beijing Superstring Academy of Memory Technology, Beijing 100176, China; miao.jiang@bjsamt.org.cn (M.J.); mingyi.yao@bjsamt.org.cn (M.Y.); ganlin.song@bjsamt.org.cn (G.S.); yuxing.zhou@bjsamt.org.cn (Y.Z.); 2 Institute of Microelectronics of Chinese Academy of Sciences, Beijing 100029, China; sujiani@ime.ac.cn (J.S.); qiyuejing@ime.ac.cn (Y.Q.)

**Keywords:** alignment, alignment marks, overlay, WQ, APD

## Abstract

With the growing adoption of 3D stacked memory structures, precise alignment and overlay control have become critical for multi-layer overlay accuracy. The metrology accuracy and stability of alignment marks are crucial to ensuring optimal alignment and overlay performance. This study systematically investigates the contributions of two key alignment parameters—Wafer Quality (WQ) and Alignment Position Deviation (APD)—to the alignment model residue in 3D structures. Through experimental and simulation approaches, we analyze the interplay between WQ, APD and overlay performance. Results reveal that APD exhibits a stronger correlation with uncorrectable model residue, particularly under global process variations such as etch non-uniformity. Furthermore, APD sensitivity varies directionally (X/Y direction marks) and spatially (wafer edge versus center), highlighting the need for targeted mark designs in process-sensitive zones. These findings provide actionable insights for optimizing alignment strategies, mark designs and process monitoring throughout R&D, technology development and high-volume manufacturing phases.

## 1. Introduction

With the continuous advancement of integrated circuit manufacturing processes, particularly in the design of advanced memory products, the application of 3D stacked structures has become increasingly widespread, leading to a further accuracy requirement for overlay control in multi-layer stacking structures. Studies have shown that alignment accuracy plays a significant role of the total overlay accuracy budget [1,2]. Therefore, optimizing the key parameters of alignment marks under 3D structures and quantifying their impact on alignment model residue has become essential for improving process robustness and overlay accuracy. Among all the alignment parameters, Wafer Quality (WQ) and Alignment Position Deviation (APD) are the two key factors, describing the contrast of alignment signal and alignment position error. This paper focuses on 3D stacked memory structures and systematically analyzes the contribution of WQ and APD to the alignment model residue through a combination of experimental and simulation methods.

WQ is a quantified indicator of the signal intensity of alignment marks captured by the alignment sensor. In lithography systems, as defined in Formula (1), WQ is typically defined as the ratio of the diffraction efficiency of a specified optical order diffraction light collected by the alignment sensor to the first-order diffraction efficiency of light with the same wavelength on a fiducial reference alignment mark [3]. Here *WQ_m_* refers to the wafer quality of the *m*th-order diffraction light, and *η*_(1,*fiducial*)_ refers to the diffraction efficiency of the first-order diffraction light on the fiducial reference mark. A high WQ value indicates that the mark signal exhibits high clarity, low noise interference and strong spatial consistency, enabling precise identification and positioning by the alignment system. Conversely, a low WQ value may lead to signal blurring or distortion, increasing the risk of alignment errors.

(1) $${WQ}_{m}=\frac{\eta_{m}}{\eta_{1,fiducial}}\;\;$$

The WQ value is influenced by a complex integration of multiple factors, such as mark design strategy (mark type, mark segmentation, segment pitch/CD and mark trench depth), process film stack (film refractive index value, absorption value and thickness) and measurement strategy (wavelength, polarization etc.) [4]. Considering the impact of process variation on WQ, it is necessary to quantify the sensitivity of WQ changes induced by different process parameters. Since all the process parameters are defined with proper target values and specs, the normalized difference WQ values can be compared when a specific process parameter changes from lower 200% to upper 200% of its specification limits. In this paper, we defined a formula to characterize WQ sensitivity regarding different incoming process impacts using the root mean square of WQ variation across all four wavelengths as in Formula (2), where *WQ_Max_*__*n*_ refers to the maximum WQ value of nth-wavelength light.

(2) $$Sensitivity=\sqrt{\frac{\sum_{n=1}^{4}{\left({WQ}_{Max\_n}-{WQ}_{Min\_n}\right)}^{2}}{n}}$$

APD reflects the measurement error of alignment mark position [5]. The source of APD is complicated, as multiple factors, including scanner platform (stage movement accuracy), alignment sensor accuracy (wavelength, polarization, etc.), mark imaging (segment, pitch) and incoming process (film stack, profile), are all possible sources for the measurement error [6,7,8]. In this paper, we focus on the mark design and incoming process impact on APD.

**Figure 1:**
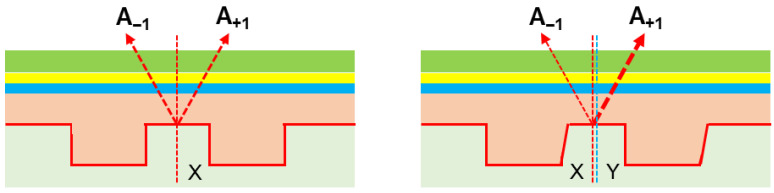
The measured error caused by an asymmetric profile of the alignment mark.

In alignment sensor systems, zero-order light is blocked, causing +1 order (A + 1) and −1 order (A − 1) signals to interfere and create intensity signal I. The alignment position is then determined through the phase of Fourier transform of the interference signal of A − 1 and A + 1, as shown in Figure 1 below, assuming the true position of the mark is X, which is independent of film stack, wavelength (WL) and polarization (Pol). In an ideal case, the mark is fully symmetric, with no magnitude or phase difference between A + 1 and A − 1. However, in practical cases, the mark is affected by the incoming process, which causes asymmetry between the left and right profile (for X direction marks), resulting in a phase difference between A − 1 and A + 1. The relationship between the phase difference (Δ*φ*) and the optical path difference (Δ*L*) is shown in Formula (3), where *λ* is the wavelength of the incident light.

(3) $$\Delta \varphi =\frac{2\pi }{\lambda }\;\Delta L\;\;$$

The alignment sensor utilizes a phase grating strategy, where the optical path difference Δ*L* is linearly related to the measurement error d. Since the wavelength *λ* and grating pitch *P* are fixed for the selected sensor and mark design, a linear relationship exists between the phase difference Δ*φ* and the measurement error d. When incident light interacts with the grating, multiple diffraction orders are generated, with the phase of each order depending on the grating’s position. Therefore, m is used to represent the diffraction order. As shown in Formula (4), the measurement system completes the transformation from phase difference to placement error, which is identified as APD here [9,10].

(4) $$d=\frac{\lambda \;\Delta \varphi }{4\pi \;m}\;\;\;\;\;\;$$

In most of the cases, the measured error cannot be fully modeled before exposure, leading to an alignment error that translates to overlay impact. Apparently, this asymmetric profile of the mark mainly comes from the incoming process, typically during etching and CMP steps [11,12].

However, quantifying the APD caused by mark profile asymmetry impact in a production line is not so straightforward. Due to the complexity of factors influencing APD, it is significantly challenging to isolate the impact of mark asymmetry on APD from all measured errors. A common industry practice is to calculate the color-to-color (C2C) APD, which involves measuring the measured error under different wavelengths of incident light and then computing the root mean square (RMS) of all measured error values across all wavelengths. Color-to-color APD combines the measurement error from different wavelengths, making it a good indicator of how sensitive the mark is to the incoming process change, but a high C2C APD may not end up with an inaccurate overlay residue, as the actual alignment strategy can still use single-wavelength color for recipe setting. Therefore, the individual APD values for different wavelengths remain key for alignment optimization, as a process-insensitive and robust mark design is always needed.

Computational alignment mark design is widely used in advanced nodes to predict alignment signal strength and overlay impact. ASML (Veldhoven, Netherlands) developed D4C software to support mark design and alignment recipe setup [7]. In our previous study, a rigorous coupled wave analysis (RCWA) method proved to be effective on WQ simulation, even in a 3D structure formed by a lateral etch process [13]. So, the same algorithm was used to simulate the incoming process impact on WQ and APD.

In this paper, we focus on 3D stacked memory structures and analyze the performance for both WQ and APD, discussing the correlation with the alignment model residue through a combination of experimental and simulation methods.

## 2. Experimental Design

**Figure 2:**
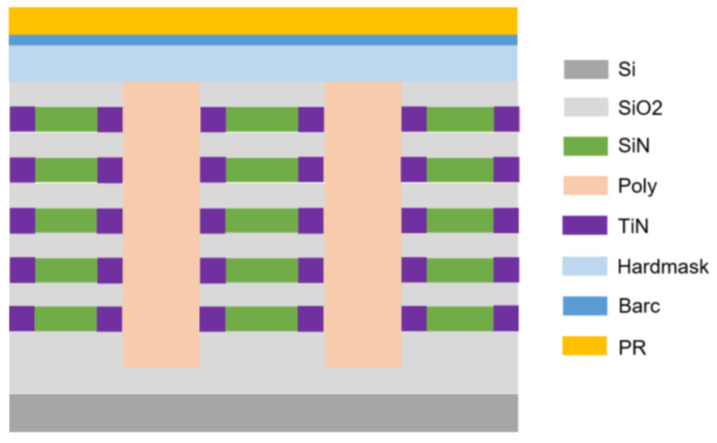
Film stack setting for the 3D structure experiment.

The film stack was designed based on a reference of a typical 3D stacked memory structure, as shown in Figure 2 below. Five tiers of SiO_2_/SiN film stacks on a SiO_2_ substrate were chosen for the incoming material. A dry etch process was set up to form NSSM53 marks, followed by a lateral etch process to partially remove SiN material. To enhance the alignment signal, a TiN thin film was deposited inside the lateral etched recess, and then the entire trench was filled with poly material, followed by a CMP process to clean up the uneven top poly layer.

**Figure 3:**
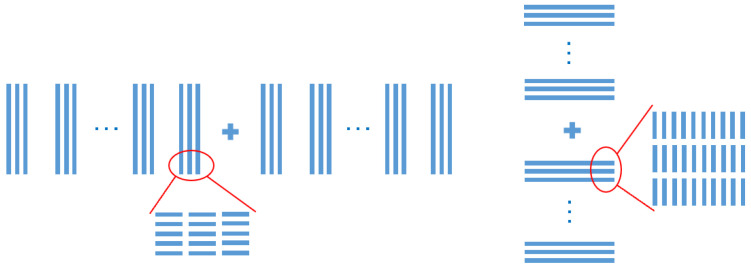
Scheme of the NSSM53 mark and cross-section of the wafer mark structure.

Considering the device’s matching requirement between the alignment marks and the array patterning, a segmented mark based on NSSM53 design was chosen as the experimental mark, which could be easily broken down into small segments. The principle of this segment design is Y-direction segmentation in the X mark with X-direction segmentation in the Y mark, as shown in Figure 3 below.

The alignment signal was collected with an ORION alignment sensor with four colors and X/Y polarization. The wavelength and polarization of incident light for both the ORION sensor and SMASH sensor are listed in Table 1 below. Wafer quality and alignment position deviation data were read out from the sensor, while mark model residues were calculated based on a full wafer-level 3rd-order polynomial model (HOWA3), which was proven to be an effective model strategy for wafer-level distortion [14,15].

The simulations were performed with the RCWA algorithm with four incident wavelengths and polarization, matching the ORION settings with wafer data. Finally, the on-wafer data and simulation data were compared to discuss the actual overlay impact.

## 3. Experimental Results

### 3.1. Polarization Selection

**Figure 4:**
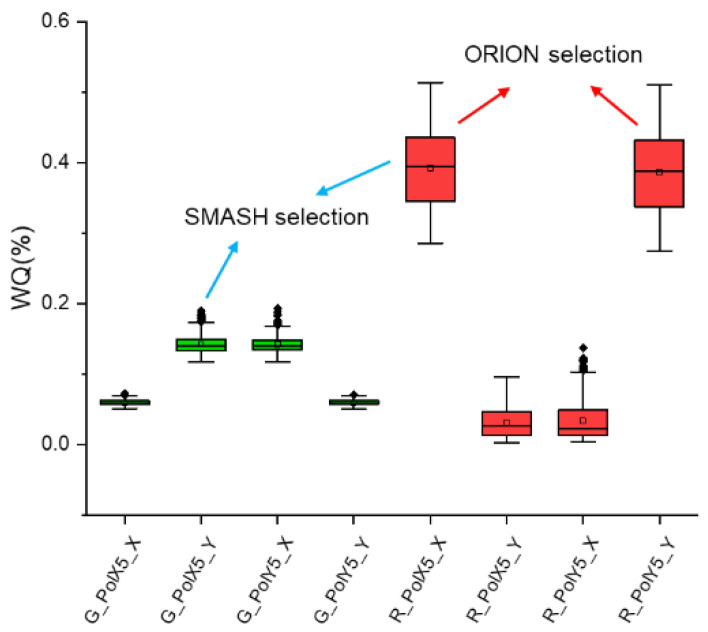
WQ of green and red colors for all polarizations.

In this experiment, a red and green color dynamic was chosen as the initial alignment strategy during exposure. The applied wavelength and polarization were decided by the WQ value, which means only the best WQ for red/green and the polarization combination was used for mark position calculation. As shown in Figure 4 below, when the ORION sensor was used, Red Y Pol for the Y mark and Red X Pol for the X mark were selected to calculate the modeled alignment grid, with a (2 nm, 1.6 nm) value of ROPI (Residue Overlay Performance Indicator) based on the HOWA3 mode. With the same dataset, the SMASH sensor would have yielded a higher value (2 nm, 2.6 nm) of ROPI, as the Y mark had to choose Green Pol Y only due to a hardware polarization limitation, as mentioned in Table 1. This example gives a very good demonstration of why the ORION sensor is naturally better than SMASH even using the same wavelength.

**Figure 5:**
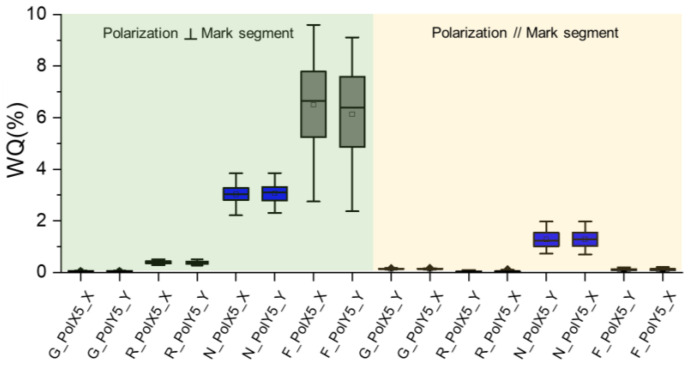
WQ of all colors broken down by mark segment and polarization direction.

From the initial data above, we can observe that the WQ value is quite correlated with wavelength, mark direction and polarization. Among all the parameters, polarization should be the first to be decided, as the mark direction and segmentation are already fixed. So, a full WQ breakdown of mark segmentation direction and polarization is listed, as shown in Figure 5 below.

In our previous study, we discussed polarization direction vs. alignment mark segment design [16]. In most of the segmental alignment mark cases, polarization perpendicular to the mark segment direction yielded a better WQ than parallel polarization. A similar observation was also shown in this experiment, as shown Figure 5 below, but this is not always the case, as the green color showed the opposite result even though the WQ value was quite low. In the next sections of this paper, we assume that the best polarization has been selected for each wavelength.

### 3.2. Alignment Key Parameter Analysis

The key alignment data were collected with a full lot, including WQ, APD and module residue, and the correlation of these key parameters is further analyzed below.

**Figure 6:**
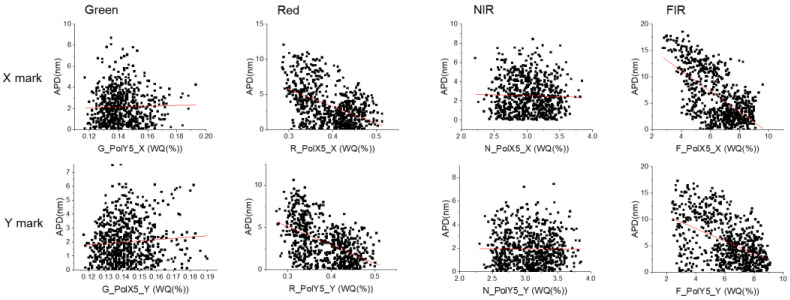
Correlation between APD and WQ for X/Y marks.

When the best polarization is confirmed for each mark and wavelength, a positive correlation can be observed between WQ and APD at certain wavelengths, as shown in red line in Figure 6 as linear fit: higher WQ samples show a smaller APD for red and FIR (but not for green and NIR), and this phenomenon shows a clear correlation with higher WQ. Theoretically speaking, WQ is impacted by mark design, process film stack and CD/pitch value, while APD mainly comes from mark profile asymmetry, so there should not be a fixed correlation between both parameters, indicating that the process variation in this case leads to significant instability for both WQ and APD simultaneously. This will be further discussed in the simulation part.

**Figure 7:**
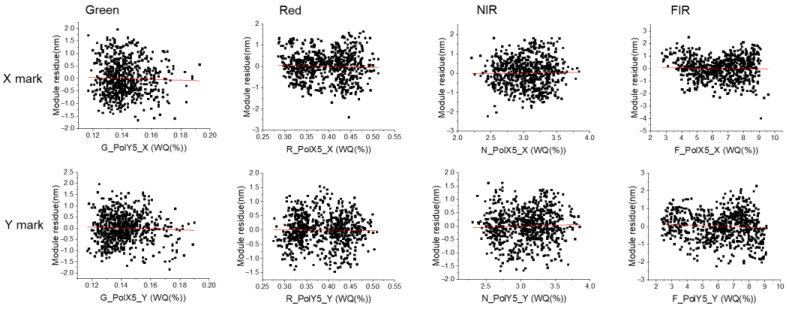
Correlation between module residue and WQ for X/Y marks.

The module residue correlation with WQ and APD was also plotted for comparison, as shown in Figure 7: the red line of linear fit is almost flat. For all the mark directions and wavelengths, there is no correlation between WQ and module residue. It can be understood that a higher WQ value gives a more robust alignment signal, but from an overlay perspective, WQ is not the most important parameter to improve the overlay accuracy.

**Figure 8:**
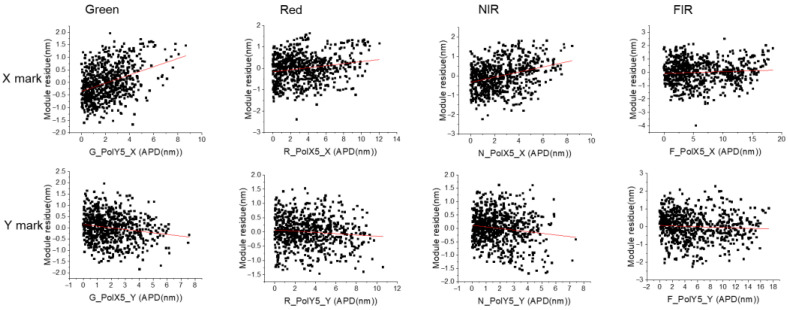
Correlation between module residue and APD for X/Y marks.

**Figure 9:**
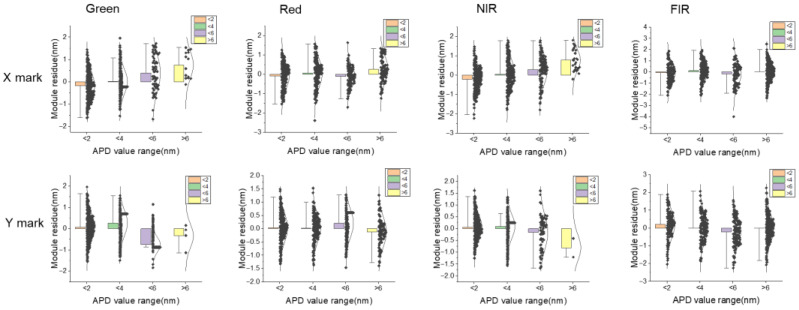
Correlation between module residue and APD for X/Y marks.

With the same dataset, a higher APD value gives a more scattered performance of the module residue, as shown in Figure 8, the red line of linear fit is tend to off center(0). So, it is clear that when the APD value shows a large variation on the same mark across the whole wafer, then after HOWA3 grid modeling, the uncorrectable model residue also shows a scattering performance. Figure 9 gives a clearer correlation of model residue and APD, with different APD value ranges defined. Note that the best model residue performance is located in the 2–4 nm APD range but not the 0–2 nm range. This is because HOWA3 modeling needs to consider all mark position error across all wafer positions, so to compensate the high position error marks, those low APD marks can be over-corrected.

Above all, the data analysis showed a clear direction of alignment strategy for overlay improvement: maintaining WQ at a reasonable performance to prevent process variation while minimizing APD variation as much as possible.

### 3.3. WQ Impact Factors

From Figure 5 above, it can be observed that the WQ value is quite wavelength dependent, as a significant difference is observed for color-to-color WQ. From our experimental data, wavelengths like FIR and NIR colors with high WQ values also showed greater variation across the wafer. From the conclusion of the previous section, the WQ value may not directly impact overlay accuracy when the signal is strong enough to capture, but it is still valuable to investigate which key process parameters cause WQ variation in this 3D structure, as these can provide a suggestion for mark design strategy.

**Figure 10:**
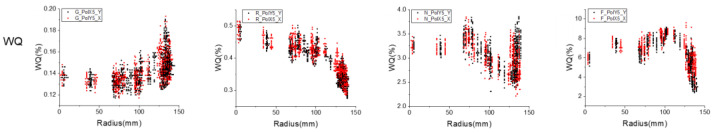
The WQ performance plot by wafer radius.

From the WQ plot by wafer radius shown below in Figure 10, all the wavelengths show substantial variation up to wafer radius change, indicating that global process variation is a dominant factor for WQ.

**Figure 11:**
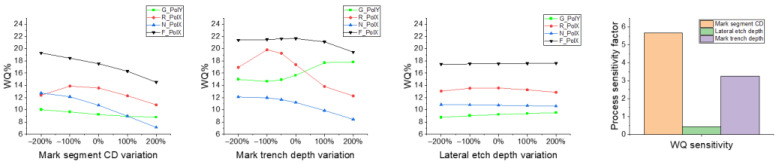
Simulation of WQ impact from etch process variations.

Here, a simulation is performed to identify the key process variations that impact WQ, including mark segment CD, mark trench depth and lateral etch depth. The film stack and mark design strategy are followed as shown in Figure 2 and Figure 3 mentioned in Section 2, and the RCWA simulation method follows that in our previous study [13]. Based on the pre-defined process target and spec, the normalized WQ value was simulated when the process parameter changed from below 200% to above 200% of its specification limits. Then, the process sensitivity of WQ is calculated with Formula (2), and all the data are shown in Figure 11 below. For this 3D structure designed as in Figure 2, the lateral etch amount and mark trench depth have less critical impacts than that segment CD.

**Figure 12:**
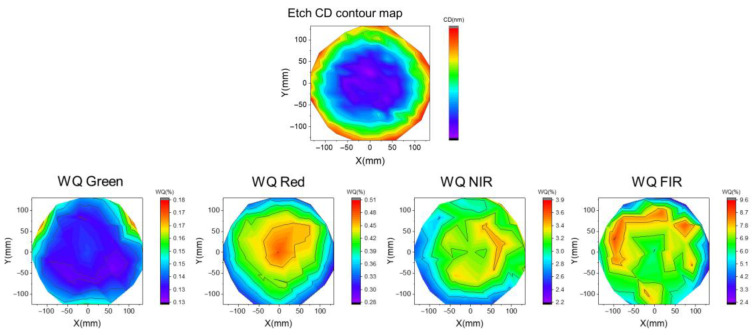
Incoming etch CD and WQ performance contour maps.

An inline full-map post-etch CD was measured with CDSEM, and a contour map was plotted accordingly in Figure 12 with a quite uniform radial symmetry map. For WQ contour maps, we do see center-to-edge differences in WQ performance with a zonal distribution for all wavelengths. Green was less sensitive regarding radius change, and red exhibited better radial uniformity. NIR and FIR showed zonal differences but less uniformity, which could be due to the impact of other process factors. Overall, the radial map comparison between CD and WQ still indicates CD’s value as a critical factor to enable a proper WQ value during alignment. Conversely, it could also be possible to use WQ to monitor incoming CD variation.

### 3.4. APD Improvement Investigation

**Figure 13:**
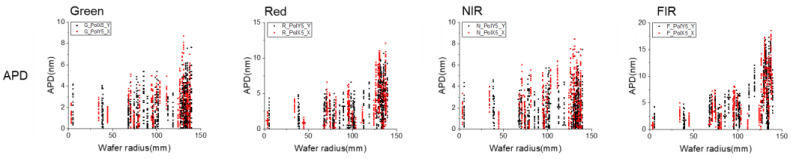
APD performance by wafer radius.

**Figure 14:**
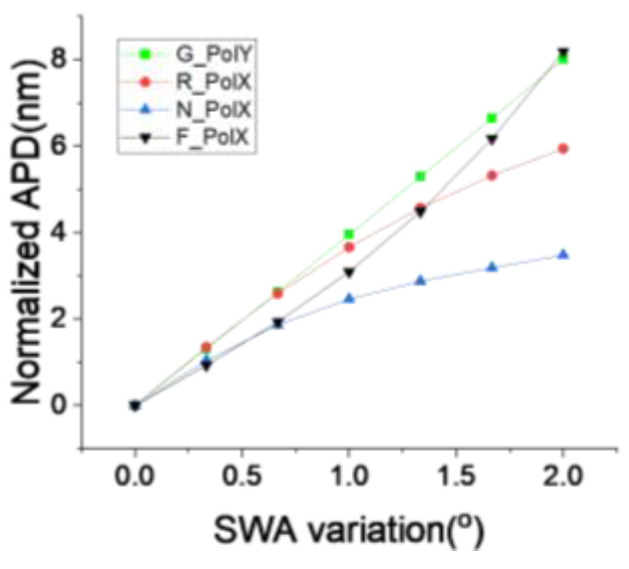
Simulation of APD impact by SWA variation.

Having clarified the correlation between APD and module residue, let us now consider how to improve module residue by reducing APD, thereby ultimately enhancing overlay. Unlike WQ, APD is more sensitive to the asymmetry of the final mark profile and is therefore less influenced by factors such as the film stack design. The main source of asymmetry in the mark profile originates from the semiconductor manufacturing process, especially etching and CMP. In the process settings used in this paper, the stack is a high-aspect-ratio 3D structure, where the asymmetry effect caused by deep etching is expected to have a significantly greater impact on the mark profile than the surface deformation caused by CMP. Variations in the etching process are typically reflected in the global wafer uniformity; therefore, we analyzed the impact of APD across the wafer radius, as shown in Figure 13. In almost all cases, APD is highly sensitive to radius changes, indicating that the primary process variation is reflected in the global uniformity. Theoretically, a pure CD change would not directly impact APD performance, while a mark trench profile side wall angle (SWA) simulation, as shown in Figure 14, shows a similar performance to inline APD, providing clear evidence that etching process-induced mark profile non-uniformity at the wafer edge is the primary cause.

**Figure 15:**
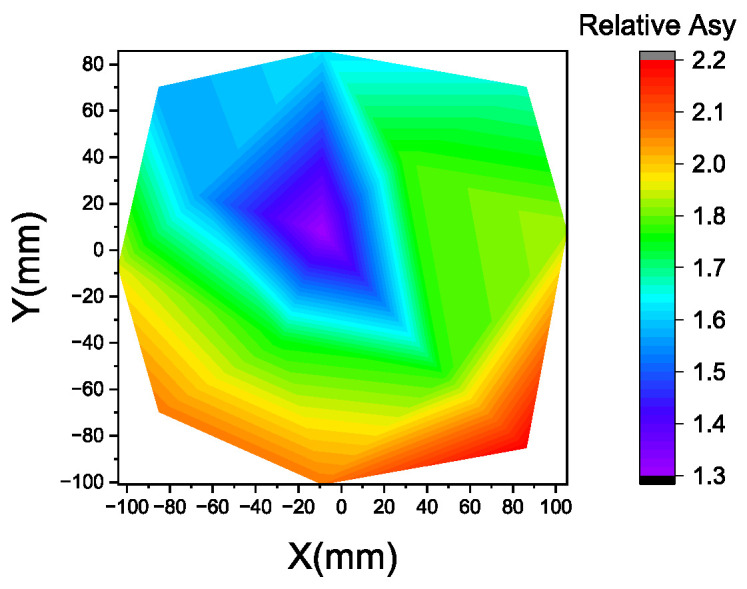
Relative asymmetry contour map for alignment marks.

Although quantifying alignment mark asymmetry across the entire wafer radius is challenging, we managed to measure sampling data points within a 100 mm radius on the wafer and calculate a normalized relative asymmetry value, as shown in Figure 15. It is not as straightforward as the etching CD contour map in the previous section, but a center-to-edge difference is still observed, and a higher asymmetry value is expected at farther wafer edge locations.

**Figure 16:**
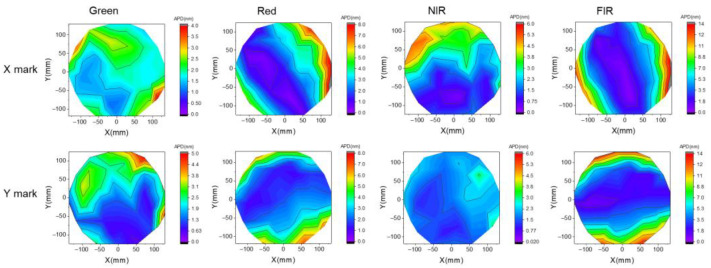
APD contour maps for X/Y marks.

All the APD data are also plotted as contour maps, as shown in Figure 16; all wavelengths show center-to-edge differences in APD performance across the inter-field layout, while a clear difference can be observed for X and Y marks. The X mark shows more sensitive APD performance closer to the wafer edge, especially for red and FIR, while the Y mark shows a similar behavior for the Y direction. This phenomenon may be correlated with the method of alignment sensor scanning through the segment marks: the ORION sensor scans through X marks horizontally from left to right, so the measurement error is more sensitive to the asymmetry profile in the X direction, even the mark is designed for Y segmentation. Conversely, when scanning Y marks vertically (top to bottom), the measurement is more sensitive to asymmetry in the Y direction, and the mark is designed for X segmentation. Although the experimental design of mark segment directions is already considered less sensitive to process changes, the asymmetry profile induced by global etch uniformity still play a key role in APD variation.

## 4. Discussion of Applications of Key Parameters

**Figure 17:**
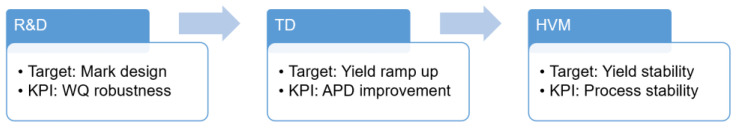
Targets and KPIs for alignment marks in different phase of a product.

In a broader perspective for the entire product lifecycle, different KPIs can be assigned during different phases of target setting. As shown in below Figure 17, different targets in Research and Development (R&D) phase, Technology and Development (TD) phase and High Volume Manufacturing phase lead to different KPIs.

During the Research and Development (R&D) phase, the first priority is always to deliver a robust mark design strategy to ensure enough contrast in the alignment mark signal, so WQ needs to be simulated with proper film stack settings. For 3D structures, it is indeed becoming more challenging, as thick and opaque hardmask material has to be chosen to enable a high-aspect-ratio etch process, which significantly weakens the contrast of the mark signal.

During the Technology and Development (TD) phase, the mark WQ should be fully qualified, so the pressure moves into overlay improvement for yield ramping up. As mentioned in the previous section, APD improvement is considered the main focus in this phase.

When the product becomes mature in the High Volume Manufacturing (HVM) phase, the overlay remains stable, and incoming process stability needs to be controlled. In this case, WQ and APD can be considered monitoring parameters for the incoming process variations. Hyunjae Cho proposed a monitoring solution for the incoming process by retrieving a wafer asymmetry map [8].

**Figure 18:**
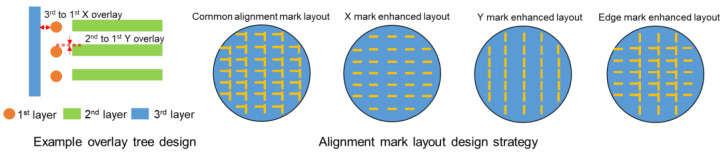
Different alignment layout design strategy.

Here, we can think of another application for a process monitoring strategy with APD. In the previous section, we noted that the sensitivity of APD vs. process shows significant direction-dependent performance: X/Y marks show more sensitive individual APD performance when closer to the wafer edge (process variation zone), and mark segmentation is already designed to minimize the process impact. For a specific device design example, as shown below in Figure 18, second-layer-to-first-layer overlay requires sensitivity in the Y direction, with less requirement in the X direction, while third-layer-to-first-layer overlay requires sensitivity in the X direction and has almost no requirement in the Y direction. This case requires different overlay monitoring strategies for the two layers. Therefore, a dedicated X/Y mark layout can be set up at individual layers to monitor specific process variation for different directions. Similarly, if process variation is only sensitive at the wafer edge, an edge-enhanced mark layout also can be designed to monitor the edge zone area.

## 5. Conclusions

This study systematically investigates the critical roles of Wafer Quality (WQ) and Alignment Position Deviation (APD) in determining overlay accuracy for 3D stacked memory structures. WQ sensitivity is defined to quantify the impact of alignment contrast from different process variations. Key process variation factors are investigated through a combination of experimental characterization and rigorous simulations. This work establishes a holistic framework for optimizing alignment accuracy in 3D structures, emphasizing APD-driven overlay control and process monitoring. The directional and spatial insights into APD sensitivity provide a roadmap for next-generation mark designs in advanced semiconductor nodes. WQ, APD and process stability are identified as different alignment mark tuning strategies in R&D, TD and HVM phases. We also propose an application of the process monitoring strategy with APD, utilizing the different APD sensitivities in the X/Y mark directions.

## Figures and Tables

**Table 1:** Wavelength and polarization of incident light.

Incident Light	Wavelength	Polarization (Orion)	Polarization (SMASH)
Green	532 nm	X/Y	Y
Red	633 nm	X/Y	X
Near infrared (NIR)	780 nm	X/Y	Y
Far infrared (FIR)	852 nm	X/Y	X

## Data Availability

The original contributions presented in the study are included in the article, and further inquiries can be directed to the corresponding author.
